# Learning of Chunking Sequences in Cognition and Behavior

**DOI:** 10.1371/journal.pcbi.1004592

**Published:** 2015-11-19

**Authors:** Jordi Fonollosa, Emre Neftci, Mikhail Rabinovich

**Affiliations:** 1 Biocircuits Institute, University of California, San Diego, La Jolla, California, United States of America; 2 Institute for Bioengineering of Catalonia, Barcelona, Spain; 3 Department of Cognitive Sciences, University of California, Irvine, Irvine, California, United States of America; Indiana University, UNITED STATES

## Abstract

We often learn and recall long sequences in smaller segments, such as a phone number 858 534 22 30 memorized as four segments. Behavioral experiments suggest that humans and some animals employ this strategy of breaking down cognitive or behavioral sequences into chunks in a wide variety of tasks, but the dynamical principles of how this is achieved remains unknown. Here, we study the temporal dynamics of chunking for learning cognitive sequences in a chunking representation using a dynamical model of competing modes arranged to evoke hierarchical Winnerless Competition (WLC) dynamics. Sequential memory is represented as trajectories along a chain of metastable fixed points at each level of the hierarchy, and bistable Hebbian dynamics enables the learning of such trajectories in an unsupervised fashion. Using computer simulations, we demonstrate the learning of a chunking representation of sequences and their robust recall. During learning, the dynamics associates a set of modes to each information-carrying item in the sequence and encodes their relative order. During recall, hierarchical WLC guarantees the robustness of the sequence order when the sequence is not too long. The resulting patterns of activities share several features observed in behavioral experiments, such as the pauses between boundaries of chunks, their size and their duration. Failures in learning chunking sequences provide new insights into the dynamical causes of neurological disorders such as Parkinson’s disease and Schizophrenia.

## Author Summary

Because chunking is a hallmark of the brain’s organization, efforts to understand its dynamics can provide valuable insights into the brain and its disorders. For identifying the dynamical principles of chunking learning, we hypothesize that perceptual sequences can be learned and stored as a chain of metastable fixed points in a low-dimensional dynamical system, similar to the trajectory of a ball rolling down a pinball machine. During a learning phase, the interactions in the network evolve such that the network learns a chunking representation of the sequence, as when memorizing a phone number in segments. In the example of the pinball machine, learning can be identified with the gradual placement of the pins. After learning, the pins are placed in a way that, at each run, the ball follows the same trajectory (recall of the same sequence) that encodes the perceptual sequence. Simulations show that the dynamics are endowed with the hallmarks of chunking observed in behavioral experiments, such as increased delays observed before loading new chunks.

## Introduction

Sequence learning is a critical component of human intelligence. The ability to recognize and produce ordered sequences is a defining feature of the brain and a key component of many cognitive performances. Sequence learning and production is a hierarchical process, such as in speech organization, behavioral sequences, and thought processes. By segmenting a sequence of elements into blocks, or *chunks*, information becomes easier to retain and recall in the correct order [1]. Such chunking organization in memory has been investigated for more than half a century, when Bousfield formulated the idea that information-carrying items seem to be recalled in associated clusters [2], and Miller pointed out that limits in our working memory capacity for processing information necessitated the organization of items into chunks [3].

A chunk is often defined as a collection of elements having strong associations with each other, but weaker associations with elements within other chunks [4]. For example, complex motor movements are represented as a chain of subordinate movements, which are concatenated in a goal-specific fashion [5]. Behavioral visuo-motor sequence learning experiments suggest that action sequences are organized as chunks of information-carrying items [6–9]. Imaging and behavioral studies further suggest that chunking learning extends to language processing [10, 11], visual perception [12], habit learning [13], and motor skills [14–17].

Several studies provided models for chunking learning that explain some behavioral observations. For example, a model of chunking learning explains why skill improves with practice according to a power law [18]. Another example is that of competitive chunking [19], whereby a bottom-up perception process strengthens the chunks. Such computational models are informative as high-level descriptions of chunking learning, but do not incorporate temporal dynamics in a natural way. As a result, such models cannot provide principled insight into the temporal aspects of behavior. On the other hand, a dynamical systems approach naturally allows the study of temporal interactions [20], and can provide tight connections with biophysical models of neurons.

Experimental findings in imaging and behavioral studies provide the structure and dynamics of chunking in the brain at the mesoscopic level, allowing one to build theoretical models for the description of chunking in cognition and behavior [21]. These models are non-linear dynamical systems that describe the interaction of core components—or cognitive modes—participating in a specific mental function [22]. Here, we describe a dynamical model of the cognitive mechanisms for learning chunking representations of sequences. The dynamical system is based on the sequential competition between different information-carrying items that are represented as metastable states, such as saddle nodes. In the neighborhood of a saddle point, elementary volumes in the phase space are compressed along stable separatrices and stretched along an unstable separatrix. Saddle nodes can be chained such that the unstable separatrix of one node corresponds to the stable separatrix of the next node along the chain. If the compressing at the saddle node is larger than the stretching and all nodes in the chain are dissipative, the trajectories stably follow a channel [22]. Such channels are known as Stable Heteroclinic Channels (SHCs), and are argued to form the basis of sequential working memory through Winnerless Competition (WLC) dynamics [23, 24].

The WLC principle depicts itinerant dynamics whereby a “winning” state transiently dominates the network in a sequential fashion. Its function is to transform inputs (*e.g.* a task input) into spatiotemporal outputs based on the intrinsic switching dynamics of an ensemble of modes [23]. As a concrete model of WLC, we employ a generalization of the Lotka-Volterra evolutionary prey-predator model [25], known as the Generalized Lotka-Volterra (GLV) model. GLVs represent a canonical non-linear model of non-equilibrium dissipative systems [26], and is widely used to study local bifurcations of SHCs. Many other models can be written in the form of GLV after some recasting [27], and its dynamical properties are consistent with a wide range of neuron models [23, 28–30].

Extending this idea, a dynamical image of *chunking* processing is a two-layer model describing a heteroclinic chain of heteroclinic chains. Under these dynamics, one metastable state in a “chunking layer” is associated to a heteroclinic sequence in another “elementary layer” [31]. In such representation, the chunks—or groups of elementary items—are learned in the “chunking layer”, whereas the elementary items are learned in the “elementary layer”. For example in the phone number 8585342230 broken down in four chunks, 858-534-22-30, each digit in a chunk is represented by a separate elementary unit, while every group of digits is represented by a chunking unit. This way, the chunking representation is a heteroclinic chain (in the chunking layer) of heteroclinic chains (in the elementary layer). Earlier work described a similar model for the recognition of sequences of sequences [32].

Our previous work demonstrated a model of sequential spatial memory learning based on the WLC principle [33]. The dynamics was endowed with learning dynamics which led to the self-organization of WLC. To learn chunking sequences, we extend our previous model with a hierarchical neural network [21], and augment it with bistable Hebbian plasticity dynamics [34] for unsupervised learning. Unsupervised here refers to the fact that learning is self-organized: During training, no external signal other than the perceptual information enters the dynamical system.

The competitive dynamics in the cognitive network and the plasticity rules interact to learn a chunking representation of the sequence. Within each layer, the couplings in the system are initialized to a state where the network performs Winner-Take-All (WTA): the node receiving the strongest input activates and all other node are silenced. When the couplings within a layer become sufficiently asymmetric, the dynamics within that layer switch from a WTA behavior [35] to a WLC behavior. At each layer the system learns chunks of information provided by the layer below it and stores syntactical information by modifying the couplings according to the directions indicated by the perceived items. After training, the system can reproduce the entire sequence by transitioning the activity of its corresponding modes in the same order.

## Results

### Network model for sequence learning with chunks

**Fig 1:**
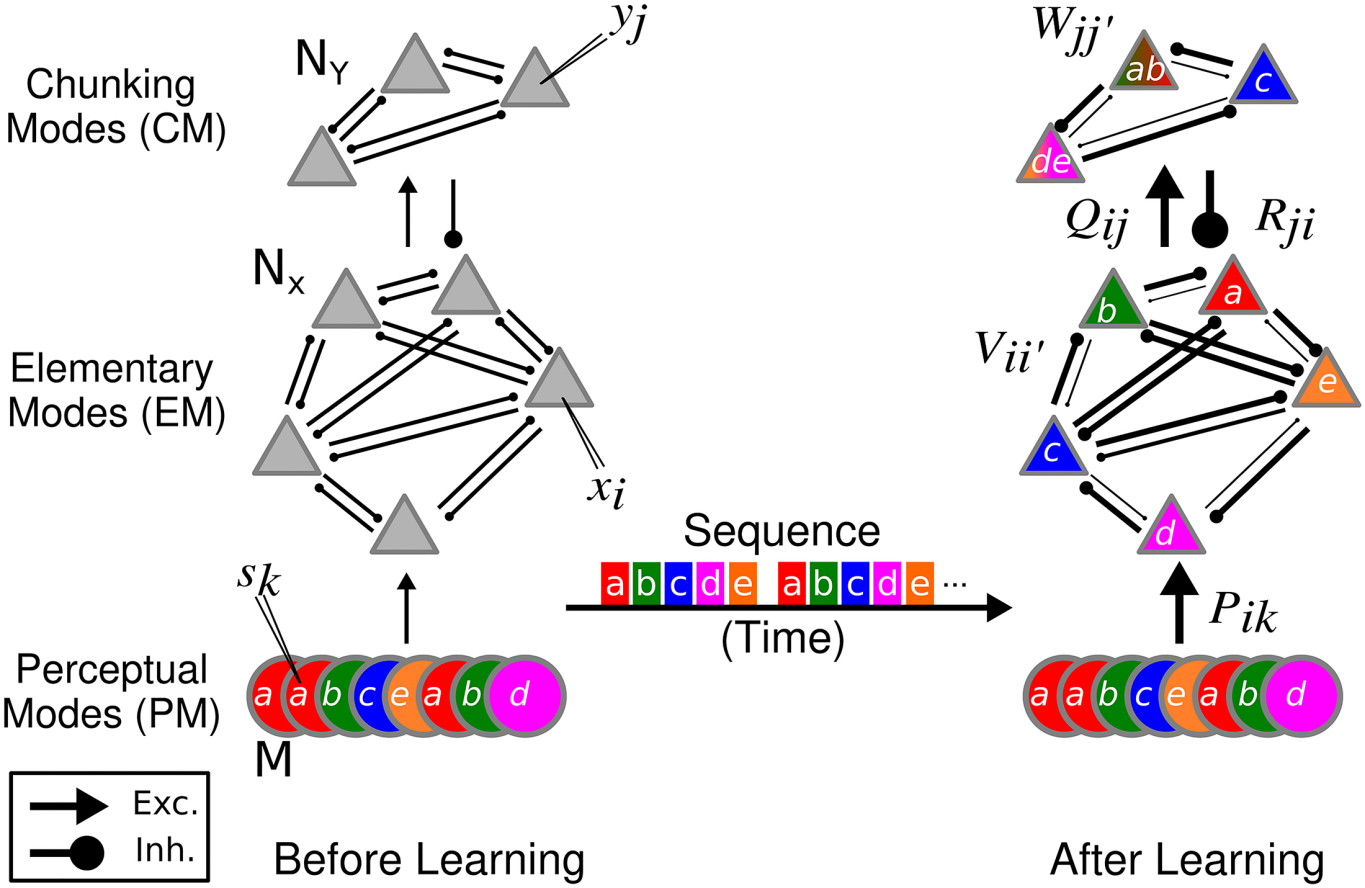
Two-layer network for learning chunking dynamics. In this example, the input sequence (a, b, c, d, e) is presented repeatedly. Initially, all the synaptic connections within a matrix are similar with small random variations. Through learning distinct elementary modes associate to each of the five patterns through weights of the projection matrix *P*_*ki*_. In the elementary layer, the weights *V*_*ii*′_ in the directions a to b, b to c, and d to e are weakened (arrow thickness denotes coupling strength), while the weights in the opposite direction are strengthened. The *W*_*jj*′_ follow a similar learning rule to three chunks: ab, c and de. Chunking, *i.e.* the information specifying the association between CM and EM, is learned in the coupling matrices *Q*_*ij*_ and *R*_*ji*_. The input in the perceptual layer is represented as non-overlapping binary patterns. For example, element a is the binary pattern **s** ^*a*^ = [11000100], input b is the binary pattern **s** ^*b*^ = [00100010], *etc*. Black circles represent inhibitory couplings, while arrowheads represent excitatory couplings. The number of elementary modes should be larger or equal to the number of patterns in a sequence. Note that there must be at least three units in each layer for a stable heteroclinic cycle to exist. It is not necessary that *N*_*y*_ < *N*_*x*_, and any value such that *N*_*y*_ > 3, *N*_*x*_ > 3 can be used. *i* = 1, …, *N*_*X*_; *j* = 1, …, *N*_*Y*_; *k* = 1…, *M*; *N*_*X*_ ≥ *M* > 3.

Our dynamical model of chunking learning is composed of Perceptual Modes (PMs), Elementary Modes (EMs) and Chunking Modes (CMs). These are organized in a two-layer network plus a perceptual input layer, as shown in Fig 1. The activity of the PMs is dictated by a pre-determined sequence of patterns, presented multiple times as a repeated loop. The PM project to *N*_*X*_ EMs, according to a projection weight matrix *P*. The *N*_*Y*_ CMs receive excitatory input from the EMs according to a weight matrix *Q* and inhibit the EMs back through a weight matrix *R*. Here, we define inhibitory as couplings that result in a negative contribution to the node activity. Within the elementary and the chunking layer, the nodes have all-to-all inhibitory couplings, the weights of which are stored in competition matrices *V* and *W*, respectively.

The two-layer chunking dynamics is a GLV system of the form:

$$\begin{array}{c} \begin{array}{cc} \tau_{x}\frac{d}{dt}x_{i}\left(t\right) & =x_{i}\left(t\right)(\sum_{k=1}^{M}P_{ki}\left(t\right)s_{k}\left(t\right)+b_{x}\\ & \;-\sum_{i^{\prime }=1}^{N_{X}}V_{i^{\prime }i}\left(t\right)x_{i^{\prime }}\left(t\right)-\sum_{j=1}^{N_{Y}}R_{ji}\left(t\right)y_{j}\left(t\right))+\sigma_{x}\eta_{i}\left(t\right),\\ \tau_{y}\frac{d}{dt}y_{j}\left(t\right) & =y_{j}\left(t\right)\left(z_{j}\left(t\right)+b_{y}\right)+\sigma_{y}\xi_{j}\left(t\right),\\ \tau_{z}\frac{d}{dt}z_{j}\left(t\right) & =-z_{j}\left(t\right)+(\sum_{i=1}^{N_{X}}Q_{ij}\left(t\right)x_{i}\left(t\right)\\ & \;-\sum_{j^{\prime }=1}^{N_{Y}}W_{j^{\prime }j}\left(t\right)y_{j^{\prime }}\left(t\right)+b_{z}\left(t\right)), \end{array} \end{array}$$ (1)

where state variables *x*_*i*_, *y*_*j*_ represent compositions of brain activities such as population firing rates [36], *b*_*x*_, *b*_*y*_ are the respective constant growth rates and *η*_*i*_(*t*), *ξ*_*j*_(*t*) are random (Wiener) processes with amplitudes *σ*_*x*_ and *σ*_*y*_ respectively. Perceptual modes *s*_*k*_ (*e.g.* visual or auditory cues) stimulate the elementary modes *x*_*i*_, which in turn drive the chunking modes *y*_*j*_ through variables *z*_*j*_. Variables *z*_*j*_ convey the regulation between different brain domains or cognitive modes [22, 37]. In our chunking model, we have used the simplest description that reminds the first order kinetic of synapses in spiking neuronal networks [38]. The *τ*_*z*_ is the characteristic time scale of *z*_*j*_ that determines the temporal distance between different informational units (*i.e.* those that would be part of different chunks) by delaying the competition between different CMs [39]. Finally, *b*_*z*_(*t*) is a time-varying bias used to dynamically modulate chunking.

We construct a dynamical learning model that concatenates sequence elements within one layer, and segments longer sequence portions in multiple groups (chunks). Such two interacting processes are believed to be at the heart of chunking learning in the brain [5, 7–9].

The key components of the learning model can be separated in two parts: 1) An asymmetric, bistable Hebbian learning rule within the WLC network learns the sequence (order) of the activity of the subordinate layer, by potentiating the weights corresponding to the transitions occurring in the elementary layer. The effect of this operation is to “concatenate” informational items, such that, during recall the same order is reproduced in a robust fashion. Hebbian learning within the WLC layer has been previously demonstrated in [33], but the proposed learning rule had a single fixed point. By selecting the two fixed points of the bistable rule according to the bifurcation of the SHC (one above the bifurcation point, one below), bistability renders the learning much more robust and prevents the formation of spurious channels. 2) The connections between two consecutive layers are learned through a symmetric, bistable Hebbian rule. This rule causes a superordinate layer to associate one (or more) modes to a group of modes in a subordinate layer. The WLC dynamics in a superordinate layer causes the network to transition its active mode, causing it to associate one mode to a finite number of modes of a subordinate layer. The association to a finite number of modes guarantees the chunking process in the learning. The number of modes within one chunk depends on the learning dynamics and the WLC dynamics in each layer. In particular we show that the size of the chunk is further bounded by the ratios of the potentiation *vs.* depotentiation magnitudes. This effect is further explained and quantified in section Learning dynamics determine chunk size.

For these two learning rules, we used the bistable rule demonstrated in [34]. This rule has been demonstrated to reproduce many of the learning curves observed in experiments, and its dynamics are well understood. Similarly to [21, 32], we can construct a hierarchy for chunking learning by setting the time constant of a superordinate layer larger than the time constant of the subordinate layer.

In addition to the learning rules above, the elementary layer learns to associate one mode to each element in the sequence through competitive learning [40, 41]. Such learning has been extensively documented and shown to perform the Expectation-Maximization algorithm [41], and is thus robust to the noise in sensory modes.

Fig 1 illustrates chunking learning before and after training. In this example, a sequence composed of five patterns symbolized as a, b, c, d, and e, is presented multiple times during the learning phase. Distinct modes associate to each of the five patterns through weights of the projection matrix *P*_*ki*_. For example, in Fig 1 the weights in the directions a to b, b to c, and d to e are weakened (arrow thickness denotes coupling strength), while the weights in the opposite direction are strengthened. The same learning dynamics apply to the inhibitory couplings between the chunking modes. In this illustration, three chunks are learned: ab, c and de.

**Fig 2:**
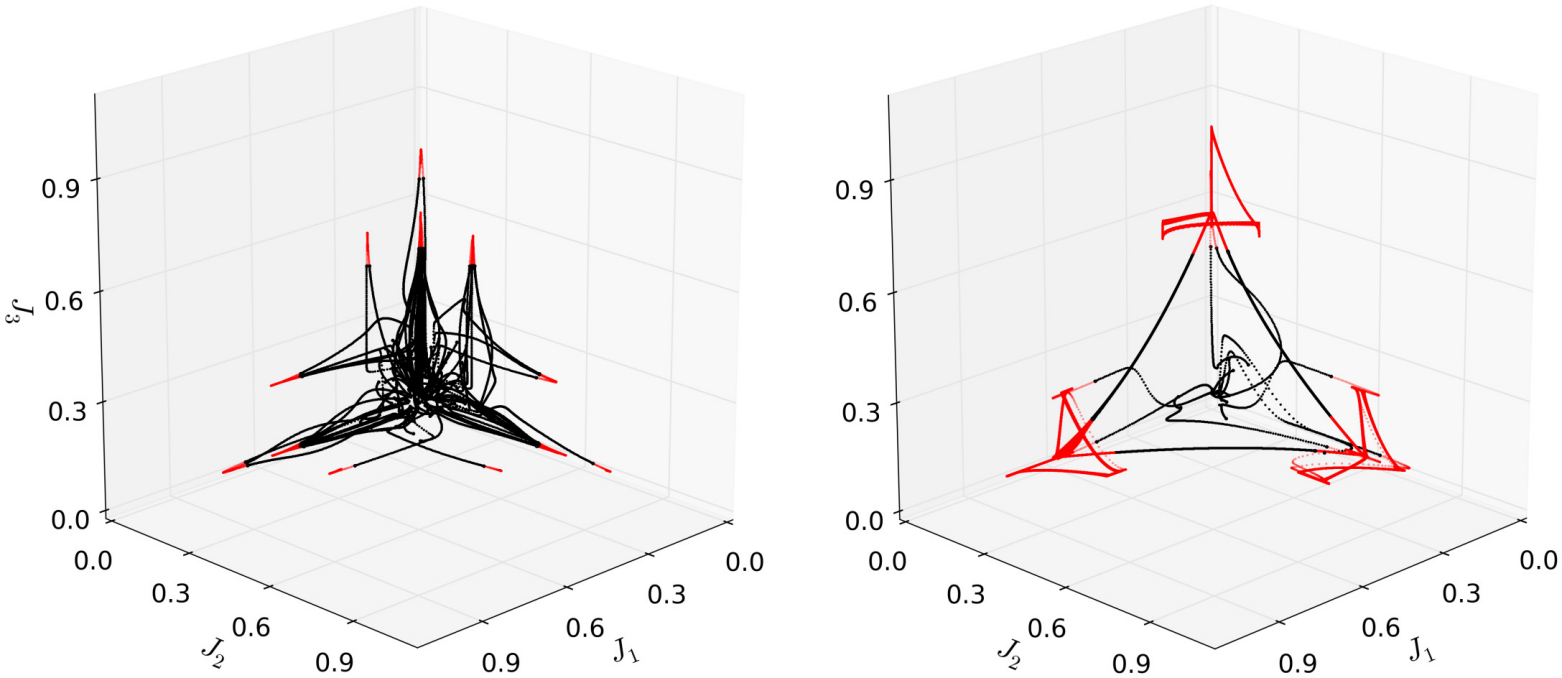
Projection of the phase portrait of the two-layer chunking hierarchical dynamics in the space of three auxiliary variables. This example illustrates the dynamics of a system *N*_*X*_ = 24, *N*_*Y*_ = 3 before (left) and after learning (right) a sequence consisting of 24 patterns of *M* = 144 pixels. For visualization purposes, the variable space was projected according to $J_{i}=.5y_{i}+.5(x_{1}^{i}+x_{2}^{i}+x_{3}^{i})$, where superscript refers to the associated chunk. The plot is colored red when either of the chunks are active (*y*_*i*_ > .9, ∀*i*). The traces were obtained from 12 runs starting from random initial conditions in the vicinity of the origin of the transformed space. Before learning, the network reaches stable fixed points. After learning, the network results in a closed chunking sequence (black) that consists of several heteroclinic cycles that represent the chunks (red). Each of the three chunks consist of EM, as the system visits the eight states in each chunk. Note however that the projection used here effectively reduces these to 9 (three states per chunk) for visualization purposes.

Fig 2 (right) shows a projection of the phase portrait of the chunking dynamics obtained after learning. Before learning, the network reaches stable fixed points, which appear as red “spikes” in Fig 2 (left). This example illustrates how learning endows the network with a closed chunking sequence (black) that consists of several heteroclinic cycles that represent the chunks, which appear as red triangles in Fig 2 (right). In general, the number of elementary items in each chunk are different and the chunking sequence can be open.

In the three following paragraphs, we detail the learning dynamics between the sensory layer, the elementary layer, and the chunking layer.

#### Association of elementary modes with sensory modes

Initially, the connections between neurons are all-to-all with random variations in their weights. The couplings within each layer are symmetric and sufficiently strong such that the network behaves as a WTA [42]. The learning in the elementary layer associates one EM with each input pattern presented in the perceptual layer, according to a correlation-based rule with synaptic scaling [40]:

$$\begin{array}{c} \tau_{P}\frac{d}{dt}P_{ki}\left(t\right)=x_{i}\left(t\right)(s_{k}\left(t\right)-P_{ki}\left(t\right)). \end{array}$$ (2)

where *s*_*k*_ are the activities of the PMs, *x*_*i*_ are the activities of the EMs. When *s*_*k*_ is stronger than the current weight, *P*_*ki*_ is increased at a rate proportional to the activity of the elementary node *x*_*i*_. Here, the negative term acts as a synaptic scaling term which prevents runaway potentiation in the weights [40]. When the inputs *s*_*k*_ are normalized, for example by feed-forward inhibition, the sum of the projection weights tends to a fixed value that is independent of the pattern [41].

#### Concatenation of sequences of elementary modes

The learning dynamics modify the weights *V*_*ii*′_ such that the order in which the EMs activate during recall is consistent with the order in the presented sequence. At each input transition, the inhibitory connections adapt such that the correct order of the presented patterns is learned in the network of elementary items. The learning rule implements a bistable Hebb rule [34]:

$$\begin{array}{c} \begin{array}{cc} \frac{d}{dt}V_{ij}\left(t\right) & \propto \alpha_{V}\left(V^{+}-V_{ij}\left(t\right)\right)\left(V^{-}-V_{ij}\left(t\right)\right)\left(V^{*}-V_{ij}\left(t\right)\right)\\ & +\left(V^{+}-V_{ij}\left(t\right)\right){LTP}_{V}\left(x_{i},x_{i^{\prime }}\right)\\ & +\left(V^{-}-V_{ij}\left(t\right)\right){LTD}_{V}\left(x_{i},x_{i^{\prime }}\right),\;\forall i\neq j, \end{array} \end{array}$$ (3)

where *V*_*ij*_ is the weight of the coupling between EMs from *i* to *j*. The first term endows the weight dynamics with two stable states, *V* ^+^ and *V* ^−^ at rest, and one unstable state V* such that 0 < *V* ^−^ < V* < *V* ^+^. The second and third terms implement the weight potentiation and depotentiation according to an asymmetric learning window (see Methods). The factors *V* ^+^−*V*_*ij*_ and *V* ^−^−*V*_*ij*_ ensure that, at rest, the weights remain in the range (*V* ^−^, *V* ^+^). When coupled with the network dynamics, an asymmetric learning window allows Long-Term Potentiation (LTP) and Long-Term Depression (LTD) to occur only when the activity transitions from one unit to another. As a result, the connection along the direction of the transition undergoes depression, while the connection in the opposite direction undergoes potentiation. The learning dynamics described above introduces asymmetry in the couplings to store the presented patterns and their order. The introduced asymmetry causes a bifurcation, changing the dynamics of the system to a WLC configuration [43, 44]. Under these dynamics, once the learning process successfully induced a WLC configuration, the state of the system moves along a trajectory composed of the saddle nodes of an underlying SHC (see Methods).

#### Segmentation of sequences of elementary modes into chunking modes

The information specifying the chunk, *i.e.* which EM belongs to which CM is stored in the coupling matrices *Q*_*ij*_ and *R*_*ji*_. Learning dynamics at the chunking layer associates CMs to groups of consecutively active EMs. The rule governing the weight updates *Q*_*ij*_ is similar to Eq (2), but with soft boundaries:

$$\begin{array}{c} \begin{array}{cc} \tau_{Q}\frac{d}{dt}Q_{ij}\propto f_{Q}\left(Q_{ij}\right)+ & \gamma_{p}^{Q}\left(Q^{+}-Q_{ij}\right)\Theta \left(x_{i}y_{j}-\theta_{p}^{Q}\right)\\ + & \gamma_{d}^{Q}\left(Q^{-}-Q_{ij}\right)\Theta \left(y_{j}-\theta_{d}^{Q}\right)\\ - & \epsilon_{H}\max(\sum_{j^{\prime }}Q_{ij^{\prime }}-m_{H},0) \end{array}, \end{array}$$ (4)

where *f*_*Q*_ is similar to the first term of Eq (3), Θ is a step (Heaviside) function and *γ*_*p*_ (*γ*_*d*_) represents the rate of weight potentiation (depotentiation). This rule dictates potentiation when both elementary and chunking modes *x*_*i*_ and *y*_*j*_ are active, and depotentiation when only the CM is active. As a result, the couplings between the pair *x*_*i*_, *y*_*j*_ are strengthened, while all the other couplings targeting *y*_*j*_ are weakened. When the number of CMs is large, the elementary modes tend to form couplings with multiple chunking modes. This causes the CM to learn chunks consisting of only one EM. To prevent this, Eq (4) includes heterosynaptic competition (last term), which imposes a limit *m*_*H*_ on the total efferent (outgoing) weights from each EM [45].

The dynamics for *R*_*ji*_ are of the form of Eq (4), but with parameters such that depression occurs when both elementary and chunking modes are active, and potentiation occurs when a CM is active. Hence, the connections from a CM to a EM that does not belong to the chunk become strongly inhibitory.

Finally, transitions between CMs are stored in the weights of the competition matrix *W*_*jj*′_, and follow the same dynamics as Eq (3).

### Sequence learning and recall

We examined the ability to learn and recall sequence of patterns of a network with the architecture described above with 3 CMs, 24 EMs and 144 PMs, as well as its ability to perform chunking. The sensory input consisted of 24 different patterns that were presented sequentially. The patterns were composed of 144 pixels that were binary for presentation simplicity. Each input pattern was composed of 6 high-intensity pixels and 138 low-intensity pixels. The high/low pixels for each pattern were selected such that there was no overlap between inputs, meaning that the position of the high-intensity pixels were different than those of the low-intensity pixels. For simplicity, we chose a stimulus that consisted of 24, non-overlapping horizontal bars. A previous analysis of the learning rule of *P*_*ki*_ showed that the shape of the patterns can be arbitrary, but the overlap and the relative sizes of the patterns increases the difficulty of the learning task [41].

**Fig 3:**
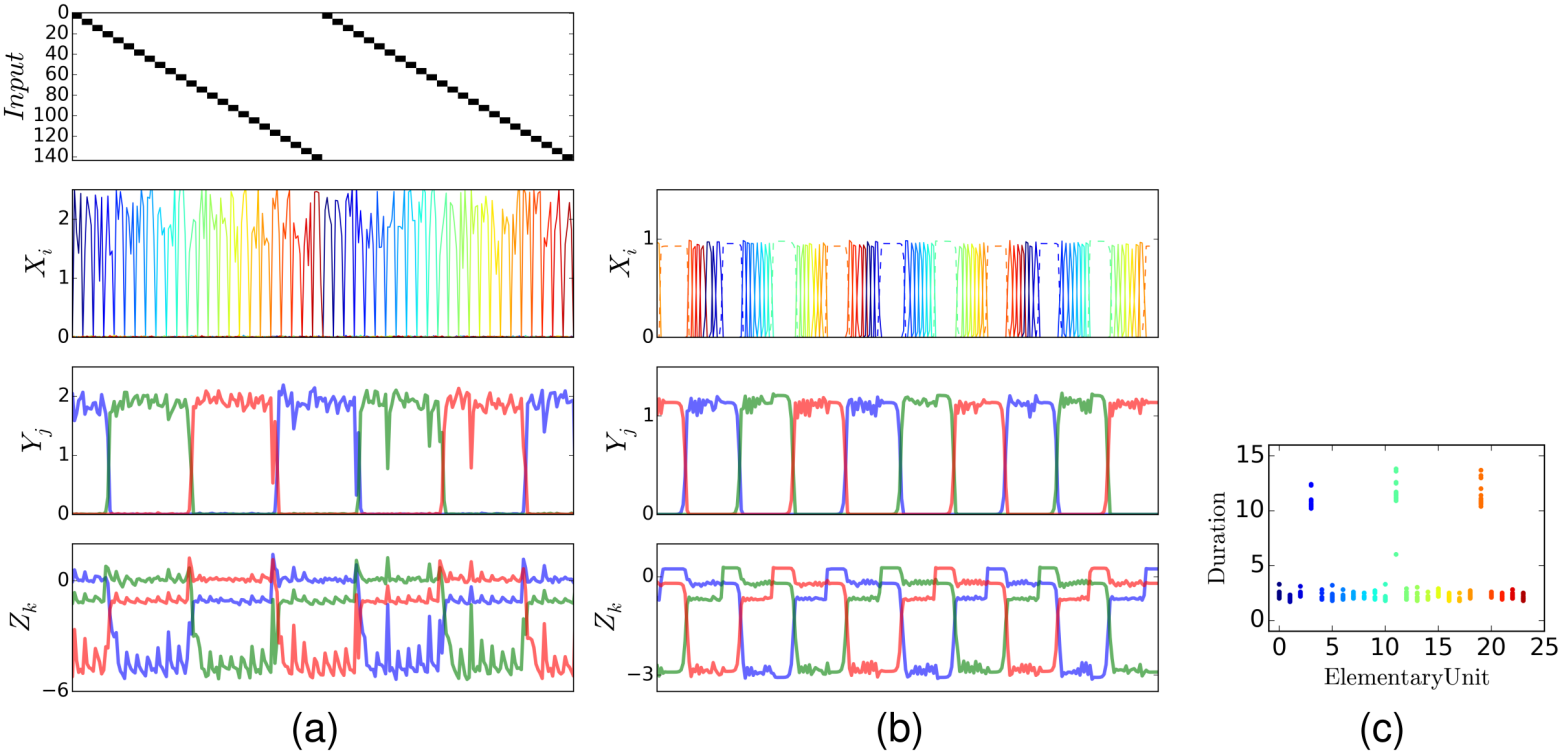
Input and network activities during learning and recall. *s*_*k*_, *x*_*i*_, *y*_*j*_, *z*_*k*_ during learning (after 5 presentations) (a) and during sequence recall (after 120 presentations) (b). Within each layer, different colors represent different modes (variables). The sensory input (presented only during learning) consisted of 24 different patterns presented sequentially. The patterns were composed of 144 binary (represented in black and white) pixels. During learning, the input drives the system dynamics. During recall, the elementary modes and the chunking modes activate in the same order as in learning. Each CM represents about 8 consecutively active elementary modes. The onset of each chunk is delayed and caused by the inhibition from the chunking layer. It is consistent with pauses before loading chunks observed in behavioral studies (highlighted in dashed line). (c) Duration that each EM remains active, with the same color codings as in (b). Three modes associated to the transitions between chunks remain active for a longer time than the others. Such pauses can be identified with pauses observed in behavioral experiments involving chunking [17].

Fig 3 shows the input patterns and the activity of the EMs and CMs during learning and sequence recall. For visualization purposes we present the activity of the PMs grouped according to their activation time.

While chunks can be formed of informational items that have some clear association with each other, chunking can also occur *spontaneously*, *i.e.* in the absence of clear structure in the stimuli [7]. In this section, we show chunking in the case of spontaneous chunking.

During the training phase, the sequence was repeatedly presented in a closed loop. After an initial transient in which EMs compete against each other, a given input pattern activates the same EM consistently (Fig 3, top). Similarly, the CMs always activate with the same subset of about 8 EMs. The resulting associations between PMs and EMs, and EMs and CMs are determined by the random variations present at the beginning of the learning. Therefore, each simulation run produced different association maps, similarly to the subject-specific chunking patterns during in behavioral experiments in the human [8].

**Fig 4:**
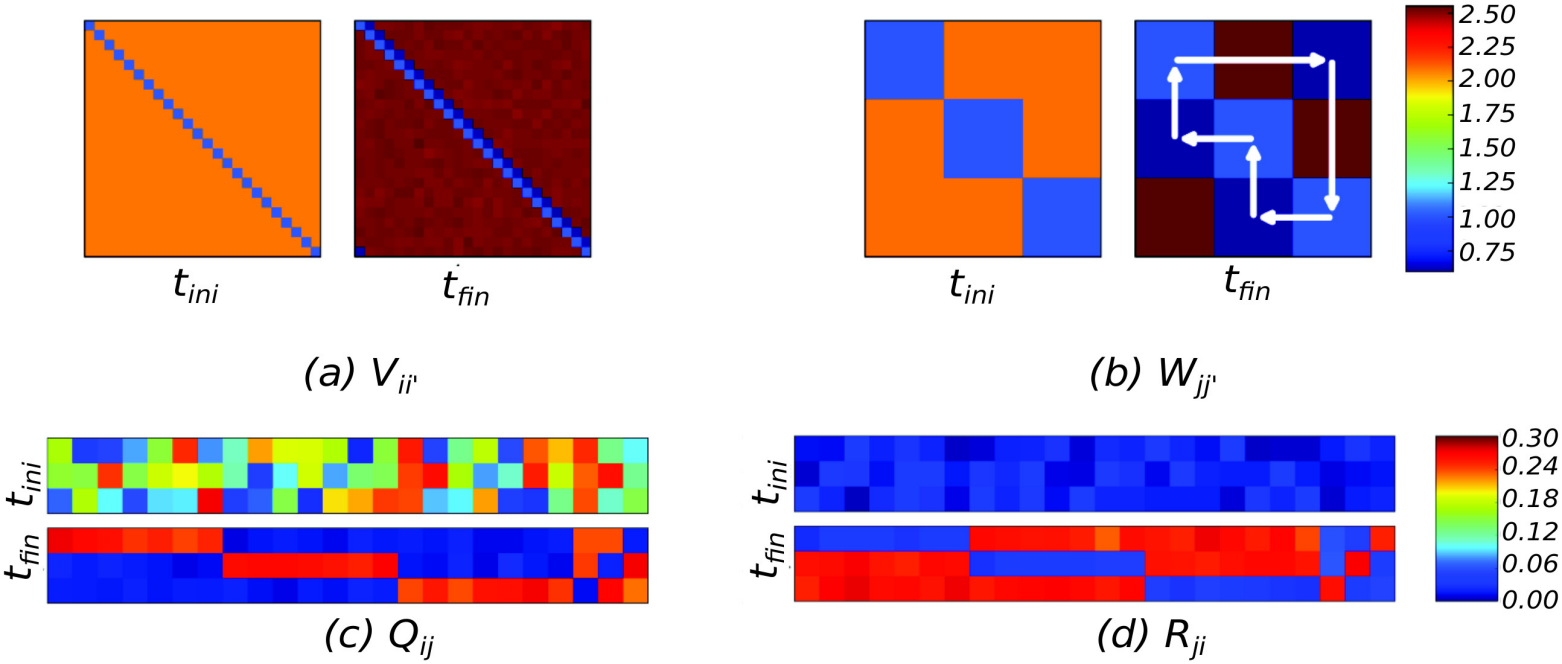
Synaptic weights before and after learning. (a, b) Initially (*t*_*ini*_), the recurrent weight matrices implement all-to-all symmetric inhibition, leading to WTA. After learning *t*_*fin*_ the matrices acquire an asymmetric component, leading to WLC. Superimposed white arrows in (b) indicate the resulting order of the recalled states. (c, d) The weights in the matrices *Q*_*ij*_ and *R*_*ji*_ learn which EM belongs to which chunk. The last three columns correspond to the elements that activate during chunk transitions.

**Fig 5:**
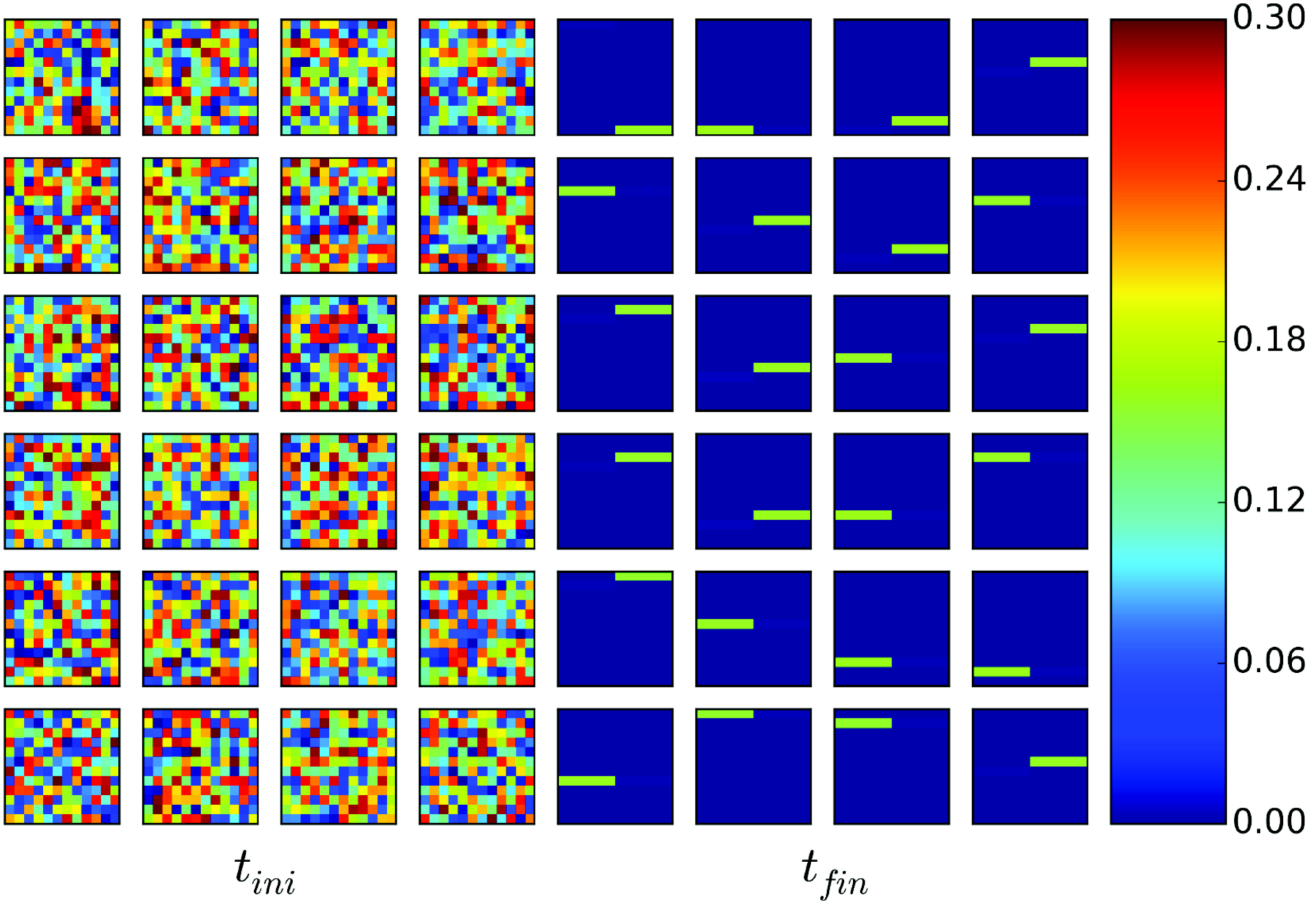
Input weights *P*_*ki*_ at the elementary modes. (left) before and (right) after training. At the beginning, *t*_*ini*_, the weights are random. The learning associates each of the 24 patterns to one EM.

After learning, the system is able to reproduce the sequence: EMs and CMs are driven with constant growth terms *b*_*x*_ and *b*_*y*_ to reproduce the activity in a periodic and continuous cycle (Fig 3, bottom). The order of the sequences were often reproduced perfectly, but the timing depends on the dynamics of the model. Namely, we observe the appearance of pauses in the EMs between chunks reminiscent of those observed in behavioral studies [7, 8]. The weights of the competition matrices, *V* and *W*, transition from a WTA configuration at the beginning of the learning to a WLC dynamics after learning (see Fig 4). Initially, the couplings are all-to-all inhibitory, leading to WTA. After learning, *V* and *W* become asymmetric, leading to WLC in both layers. The arrows in Fig 4 illustrate the succession of the state transitions in the resulting WLC. The matrices *R* and *Q* evolve to store the chunk association map. Fig 4 (Bottom) shows that weights in the matrices *Q* and *R* form three groups with similar weights which correspond to the chunks. The patterns presented to the system are stored in the synaptic weights of the projection matrix *P*. Successive presentations of the input pattern modify *P* such that the presented patterns are stored (see Fig 5).

### The Dynamics of chunking learning

The results above used a small chunking layer (*N*_*y*_ = 3) in order to illustrate the model. However, the dynamics of chunking during learning are much more interesting for a large chunking layer, since the number of possible state trajectories grows factorially with the size of the network [23]. For this reason, in the results below, we test the model for *N*_*y*_ = 30 and *N*_*x*_ = 30.

The training of the model consisted of multiple epochs. Each epoch consisted of a full sequence presentation phase, immediately followed by a recall phase. After the sequence had ended, the recall phase was initiated by cueing the network with the first element of the sequence and observing the ensuing sequence of patterns in the elementary layer. During the recall phase, the parameters of the network were kept fixed (no learning).

We quantified recall by computing the normalized Levenshtein distance between the presented sequence and the reproduced one (see Methods—Characterizing Sequence Recall). Using the Levenshtein distance, we observe that overall 95% of the elements in the sequence were reproduced.

The progress of chunking learning is monitored by inspecting the magnitude of the chunking and the presence of sequential activity in the chunking layer during recall. The magnitude of the chunking is monitored by computing the *chunking rate* during learning, defined as the number of transitions taking place in the chunking layer during the presentation of each pattern in the sequence. A chunking rate equal to 1 signifies that a different CM was active for each pattern in the sequence (no chunking), while a chunking rate significantly smaller than one during training implies that chunks were formed. Note that a measure based on sequence recall only is not sufficient to characterize chunking since accurate recall is possible without the chunking layer.

To further assess the robustness of the chunking in the presence of noise in the sensory layer, a fixed noise drawn from a rectified Gaussian distribution was independently added to each pixel at each presentation of a sequence element (see also section 3 of S1 Text). Sequence recall accuracies (measured using the Levenshtein distance) and the chunking rates degraded gracefully as the noise magnitude was increased.

We observe that the boundaries of the chunks can change from trial to trial during training, and that chunks can undergo substantial reconfigurations throughout the learning, including the creation of new chunking modes. The dynamical nature of chunking was already observed in behavioral experiments, where chunk boundaries could vary substantially even after a large number of trials [7, 46].

**Fig 6:**
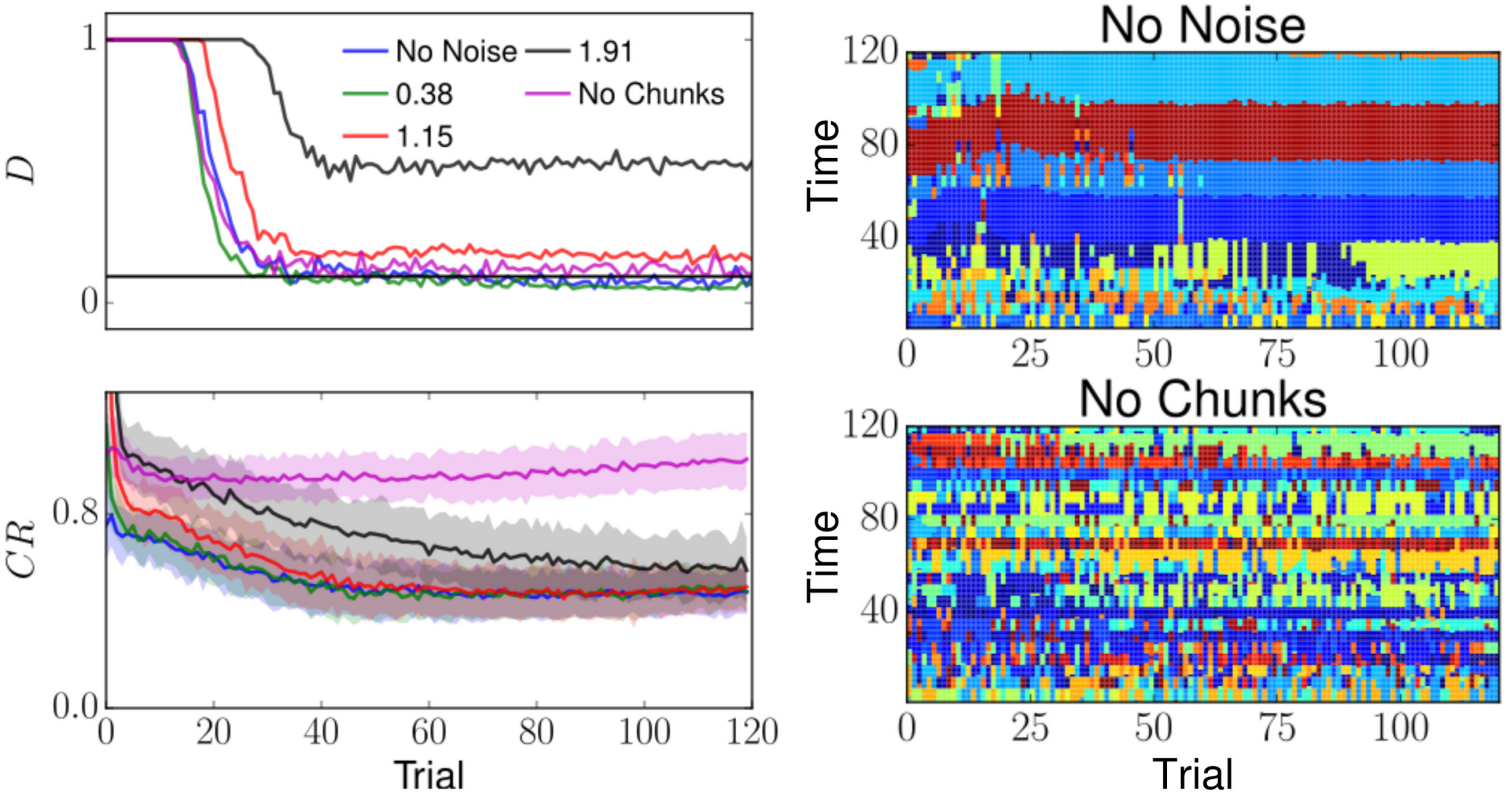
The dynamics of chunking. The model is run 60 times, for 120 trials (*N*_*y*_ = 30) for different levels of noise. Each trial consisted of the presentation of one sequence, followed by a recall phase. (Top-Left) Sequence recall accuracy *D* averaged over all the runs. The sequence was determined by the identity of the most active mode in the elementary layer.*D* was computed using the Levenshtein distance (equal to the number of additions and subtractions between two sequences). In the noiseless and low noise cases, the distance between the presented sequence and the reproduced sequence reached about.05 (horizontal line), roughly corresponding to 1 addition/subtraction per sequence recall. The network was robust to noise, and sequence recall accuracy degraded gracefully as the amplitude of noise was increased. (Bottom-Left) Estimates of chunking rate measure *CR* for monitoring chunking in the noiseless case (blue curves).*CR* is defined as the number of transitions taking place in the chunking layer during the presentation of a pattern in the sequence. During an initial transient *CR* decreases as learning proceeds, indicating the formation of the chunks. (Right) Activity in the chunking layer for two representative runs, one with no noise, the other with no chunks, where learning of *Q*_*ij*_ and *R*_*ji*_ was turned off. The identity of the chunks is color-coded. Interestingly, the boundaries of the chunks can change during training, and the chunks can undergo substantial reconfigurations at the beginning of the training phase. In absence of learning in *Q*_*ij*_ and *R*_*ji*_, the chunking rate did not diminish over the course of learning, indicating the absence of chunks. S4 Fig displays the evolution of the individual weights for the run shown in the top-right panel (No Noise).

[46] use a Bayesian algorithm combining reaction time and error rates to reveal the chunking structure in humans performing a discrete sequence production. Interestingly, the chunking structure also evolves slowly over the course of the trials. A visual inspection of our model results suggests that this slow evolution might be caused by the enrollment of new chunking modes and the disenrollment of existing ones (see Fig 6, right panel).

### Pauses in activity precede the recall of a chunk

Chunks in motor learning are often identified by the pauses between successive actions [49]. More specifically, psycholinguistic studies often focus on pauses between words and utterance-final syllable prolongations [50], which are indicative of a hierarchical organization of the overall speech production apparatus [10]. Other experiments also show the hierarchical organization of information in chunks when performing other visuo-motor tasks [5–9]. The network activity in our model exhibits a temporal structure that is reminiscent of these studies. In the recall phase, the network activity is paused until the new chunk has been “loaded” (Fig 3(c), dashed lines in Fig 3(b)). The pauses in the chunking are a result of the synchronization between elementary chunking layers. The duration of the EM and the CM activations depend on the magnitude of the growth terms *b*_*x*_ and *b*_*y*_, but the two layers are bound to each other by the feedback connections *Q*_*ij*_ and *R*_*ji*_. As a consequence, the EMs are delayed until the next chunk in the sequence is activated. The function of the pause is therefore to synchronize the activity of the CM and the sequential activity of the EM belonging to this chunk, and therefore depends on the relative speed between the elementary layer and the chunking layer. The duration of the pause is variable and did not depend on the number of items in each chunk.

In [7], the pause is assumed a direct result of two interacting processes running in parallel: one segmenting long sequential structures into shorter ones, and one process concatenating these same groups of motor elements into longer sequences. In our model, the ongoing competition within the layer and the cooperation between its layers are also two interacting parallel processes as in [7]. Concatenation in our model is performed by the competitive process along a given layer, while segmentation is performed by the cooperative couplings between layers. Our model is therefore consistent with the one described in [7].

### Learning dynamics determine chunk size

**Fig 7:**
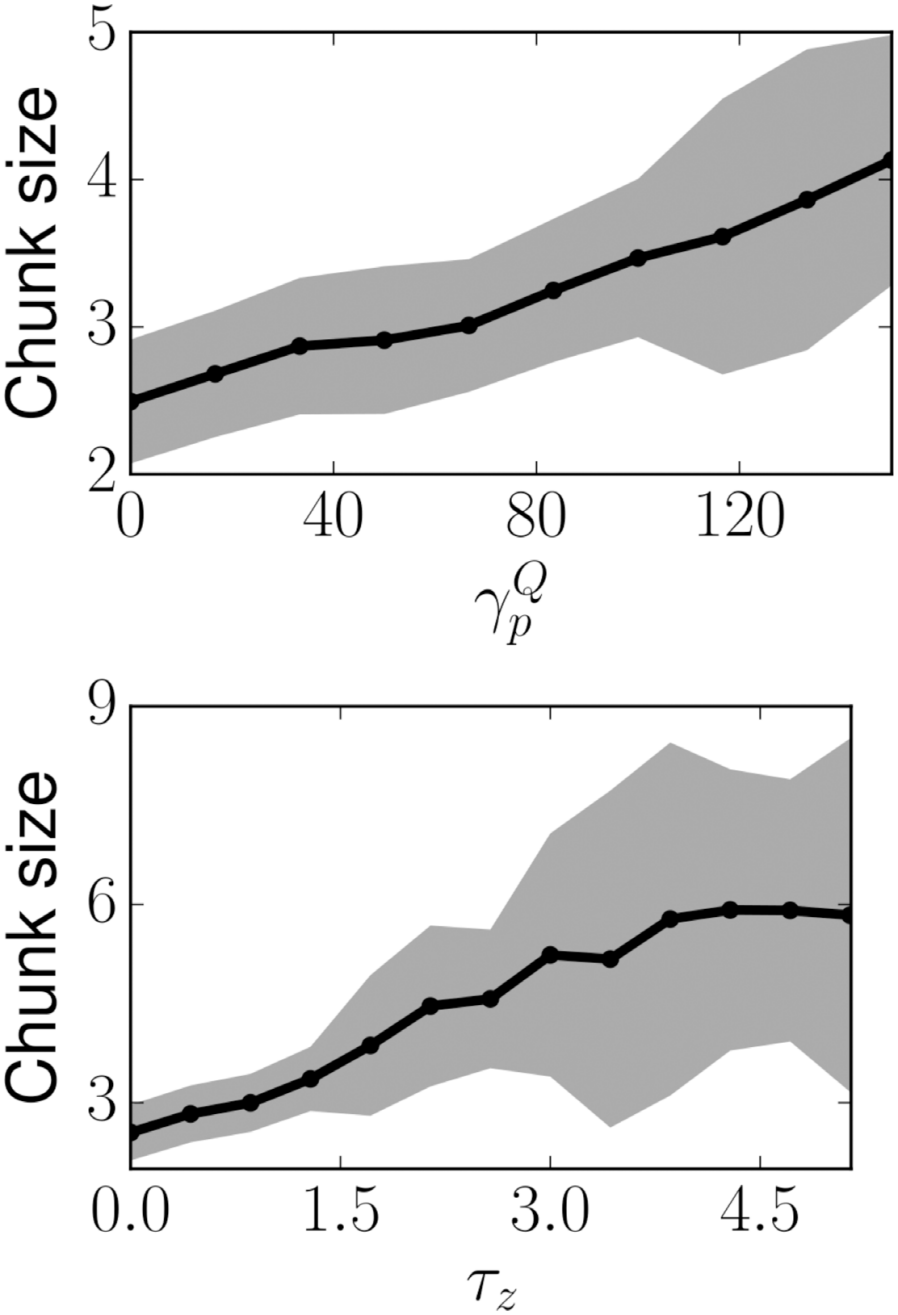
Chunk size, number of EM in each chunk, (left) as a function of the potentiation scaling factor in Q, $\gamma_{p}^{Q}$, (right) as a function of the time constant in the synaptic dynamics, *τ*_*z*_. The number of information-carrying items contained in the chunks depends on the system dynamics, suggesting that they have impact on the total capacity of the memory. The initial random conditions lead the system to different structures after learning (number and size of chunks). The case *τ*_*z*_ = 0 corresponds to completely removing the synaptic dynamics. Although the chunking is present in the absence of *z*_*j*_, the characteristic time scale of *z*_*j*_, *τ*_*z*_ has a powerful effect on chunk size. Each point was evaluated 100 times and the mean and standard deviation are presented, suggesting a monotonically increasing relationship between chunk size and $\gamma_{p}^{Q}$ or *τ*_*z*_. In total, 98.6% of the runs exhibited sequential activity in the chunking layer. Total number of available chunk modes, *N*_*Y*_ = 30; total number of elementary modes, *N*_*X*_ = 30.

In the learned state, we find that the number of items in each chunk depends on the learning dynamics and the time constant in the synaptic dynamics *z* (Fig 7). The chunk size is the result of an equilibrium between competing learning processes in the dynamics. The size of the chunk is bounded by the magnitude of the *Q*_*ij*_ and *R*_*ij*_ potentiation when *x*_*i*_ and *y*_*j*_ are co-active, and the magnitude of the depotentiation when other elements *x*_*i*′_, *i*′ ≠ *i* belonging to the same chunk are active. This is because a coupling between a CM and EM undergoes depotentiation when other EM belonging to the same CM are active. The maximum number of elements in a chunk will therefore be limited by how much a CM and a EM potentiate when both are active versus the magnitude of the depotentiation when only the CM is active (and other EMs belonging to that chunk are active). This observation suggests the important result that the neural mechanisms for acquiring the chunking sequence also play a role in determining the capacity of chunking sequential memory, and lead to new experimental predictions. For example, there is evidence that dopamine modulates the cortico-striatal plasticity chunking during motor sequence learning in humans and monkeys. In monkeys the learning of new sequences was significantly affected by injection of a dopamine receptor antagonist, but did not affect sequences that were learned prior to the injection [47]. In the context of our model, this dopamine related modulation could translate into reducing *γ*_*p*_ or increasing *γ*_*d*_. For example, if *γ*_*p*_ were gradually reduced, our model would predict a gradual decrease in chunk sizes in a chunking task such as those conducted in [7, 8] (*e.g.* Fig 7, left). Note that not all of the chunking units are used to learn and recall the presented sequence, and therefore they remain available for the learning of other sequences.

Chunk size can also be modulated within the sequence, by injecting a time-varying input into the synaptic variable *z*_*k*_. We observe that the chunk size is proportional to the magnitude of this input S2 Fig. A neural analog of this modulation can be viewed as top-down attention [48], where sequential attention switching between multimodal mental activities depend on internal or external cues.

## Discussion

Chunking is a naturally occurring process by which information-carrying items are grouped and these groups are related to each other according to a learned syntax. Chunking simplifies task performance and helps break down problems in order to think, understand, and compose more efficiently [1]. Several studies suggested that animals can effectively increase the capacity of their working memory by grouping multiple informational items into chunks [1, 3, 4, 46, 51]. Studying dynamical neural models capable of achieving chunking in a robust, scalable and efficient manner can shed light onto the organization of learning, memory and information processing in the brain.

In experimental studies, the markers of chunking are the pauses and reaction times observed during sequence production tasks. To provide a dynamical account of these studies, we presented a dynamical model capable of learning patterns and their order as metastable states of a hierarchical Stable Heteroclinic Channel (SHC). Our model provides the possible dynamical origin of delays (pauses) before a new chunk is initiated.

Recent work [21, 32] described non-linear dynamical models of the chunking process (also called sequences of sequences [32]). Rigorous analysis further confirmed that chunking behavior in their suggested model corresponds to a hierarchical heteroclinic network in phase space [31]. We propose a model that builds on [21] by introducing a synaptic weight update rule that accommodates the unsupervised learning of the chunking process.

Our SHC-based approach guarantees robustness and sensitivity, which are two critical features for information processing with transient brain dynamics. Robust transients and sensitivity to inputs may be seen as contradictory requirements. However, previous work showed that spatiotemporal modes that contain metastable states can overcome this contradiction [52–54]. In our model, the activity in the system transitions from one metastable state to another along a SHC. The topology of the corresponding SHCs is strongly dependent on the stimuli, but the channel itself is structurally stable and robust against noise [22].

To demonstrate our findings, we used software simulations of the Generalized Lotka-Volterra (GLV). The GLV model is a non-linear dynamical system that is attractive for its mathematical simplicity: the existence of a SHC can be proven rigorously [44], and in the three-dimensional case its bifurcations have been extensively investigated [43]. Furthermore, the features of the GLVs relevant to this study can be replicated in dynamical systems that describe biological processes of neurons, such as integrate & fire neurons [28], Hodgkin Huxley neurons [29], Wilson Cowan networks [30] and Fitzhugh Nagumo neurons [23].

Our model self-organizes to learn and recall sequences in a robust manner. Before learning the system has a single fixed point that depends on the applied stimulus and the initial conditions of the couplings. During training, the asymmetry in the inhibitory couplings increases and the network transitions from a Winner-Take-All (WTA) to a Winnerless Competition (WLC) configuration, such that the order in which the modes activate in the WLC is consistent with the presented sequence of patterns. Both the input patterns and their order are learned according to a hierarchical order: at a lower layer composed of elementary modes and at a higher level composed of chunking modes. When a chunk is recalled, the elementary layer incurs a pause that is similar to the delays observed at the boundaries of putative chunks observed when humans produced learned sequences [5, 7–9].

It is believed that chunking learning is a direct result of two separable interacting processes running in parallel: one segmenting long sequential patterns into shorter ones, and one process concatenating these same motor elements into longer sequences [7, 55, 56]. Our dynamical model naturally incorporates these two processes: Learning within the WLC dynamics within a layer concatenates the informational items through asymmetric Hebbian learning; while learning between WLC layers, combined with the competitive dynamics of the superordinate layer, mediate the segmentation the sequence of informational items. A direct consequence of two interacting layers are pauses in the activity: A subordinate layer is delayed until activity in the superordinate layer completes a transition.

### Capacity of the WLC network

The number of sequences that can be stored simultaneously in the network is the total number of elements in all the learned sequences, since one unit is required for a single element of a sequence. In the case of a closed SHC, the number of different sequences that the SHC can store is equal to the number of distinct channels than can be formed with *N* nodes, which is of order exp(1) ⋅ (*N* − 1)! [23]. We note however, that under reasonable neuro-biological perturbations of the recurrent connectivity, the capacity is reduced. In that case, the maximal sequence length that can be stably recalled is about 7 [57]. Our model raises new questions on chunking capacity and recall under such perturbations. The benefit of chunking can be studied by comparing the maximal length of sequence in the presence or absence of chunking. This study is complicated by the fact that the average chunk size in the network is strongly dependent on the parameters of the learning dynamics (Fig 7), and is the target of future work.

Note that for simplicity, our current model cannot learn sequences that have recurring patterns. However this is possible in principle since other closely related work dealt with recurring patterns in sequences by retaining a memory of the past patterns in the sequence [58, 59] or by using “template” connectivity matrices [32].

### Related hierarchical sequence learning models

The learning in the elementary layer of our model shares many features with models of competitive learning [60, 61] and self-organizing maps [62]. In competitive learning, each stimulus is compared with a feature vector stored at each neuron. The neuron with the highest similarity is selected as the winner, and the feature vector is updated. This mechanism is similar to the effect of learning in the projection matrix *P* and the competitive dynamics in the WLC in our model. Our model extends this idea further by embedding the order of the stimuli in the network as winnerless competition dynamics.

Our model bears strong similarities with previous work in the recognition of sequences of sequences [32, 63, 64]. Kiebel et al. study the recognition of complex sequences, where the generative model is assumed *a priori* [32]. There, the within-layer connectivity matrix is modulated by activity in supra-ordinate levels. In contrast, feedback in our model is an additive term whose effect is to turn on or off circuits (SHCs) in the subordinate layers. This modeling choice comes at the cost of more nodes, but does not require the modulation of the connections. While the model presented in [64] addressed the learning of sound sequences, it did not address the learning of chunks (*i.e* sequences of sequences).

Other related methods for learning sequences in brain-inspired models are reservoir computers [65–67], synfire chains [68–70] and chains of WTA networks [71]. The idea of exploiting asymmetrically coupled networks for sequence learning was reported in multiple works based on attractor networks [45, 58, 65, 69, 72–74]. The novelty of our approach is the learning of the hierarchical dynamics as a sequence of metastable states. Hence, our model offers a non-linear dynamical perspective on the problem of hierarchical sequence learning in neural substrates that is fundamentally different from attractor networks.

Another attempt to map this type of dynamics on the cortex is the hierarchical temporal memory model [75], although that work does not address the dynamics of biologically inspired learning of hierarchical sequences.

### Stability of the learning dynamics and robustness to parameters

Stability can be viewed from two related perspectives: robustness of the dynamics to noise in the nodes and in the connections (structural stability); and stability of the metastable states, *i.e.* their Lyapunov exponents. In either case, the study of learning stability in the general case is notoriously difficult, because the addition of new information-carrying items can destroy existing metastable states for example by creating spurious attractors [76]. In the three dimensional case, the Lotka Volterra dynamics can be thoroughly analyzed. However, many more difficulties appear in four or more dimensions, such as new metastable states in the phase space of the system, making the analysis much more difficult [36].

However, it is possible to gain some insight in the asymptotic case where the time scales in the system are well separated. In our case these are arranged such that *P* reaches equilibrium before *V*, *V* before *Q*, *W* before *R*. The overall dynamics of the elementary *P* associates stimulus items to neurons through a competitive learning mechanism and can be thoroughly analyzed. Because *P* modulates the increment to the nodes, it does not interfere with the structure of the elementary network. As long as LTP and LTD in the couplings *V* and *W* are balanced and the transitions in the network are monotonic, the weights in the network tend to a WLC configuration (see section 1 of S1 Text).

The dynamics of the synapses between EM and CM capture the chunking behavior, and are very similar to the *P* dynamics. It segments the chain of activations in the elementary layer into chunks, by detecting change points in the sequence. Its function is comparable to sequence segmentation using the sliding window algorithm commonly used for online natural language processing [77].

In this asymptotic case, the parameters can be selected manually such that learning at each time scale progresses as described above.

### Failures to recall chunking sequences

In some cases, the model failed to recall the chunking sequences, especially when the parameters of learning dynamics were not appropriately chosen. The scenarios through which recall fails is of particular interest because these can provide insights into the dynamical causes of chunking deficits in neurodegenerative diseases, such as Parkinson’s disease.

The most common cause of failing to learn was that a transition between two EM’s did not form, or was not strong enough to drive it. As a result, the state of the network remained “stuck” and is reminiscent of certain motor disorders observed in Parkinson’s patients. The recall typically resumes by providing a stimulus corresponding to an item in the cue, which is consistent with how sensory cues can improve symptoms of bradykinesia [78].

Similar behavioral observations were made on elderly who could not learn motor chunks during a sequence production task [79]. In the elderly, reduced cognitive abilities impede the learning of motor chunks, although most of the tested individuals were capable of correctly reacting to the stimuli that indicated the sequence to recall. In our model, this is equivalent to a successful learning between the perceptual layer and the elementary layer, but failing to learn the weights within the elementary layer.

In other cases where learning failed, the chunking modes did not reach a WLC configuration, although the sequential structure was learned in the elementary layer. The result is that the activity in the chunking layer remained constant and did not affect the sequential structure of the EMs activations. This shortcoming was revealed in the elementary layer by the lack of pauses during the sequence recall.

### Conclusions

In this paper, we proposed a model of hierarchical chunking learning dynamics that can represent several forms of cognitive activities such as working memory and speech construction. This model is capable of learning patterns and their order as metastable states of a hierarchical SHC, and reproduces several key features observed in chunking behavior in humans.

The model and the results outlined in this paper sheds new light onto the formation of sequential working memory and chunking. Complex action (such as speech or song production) can be viewed as a chain of subordinate movements, which need to be combined according to a syntax in order to reach a goal.

Recent studies suggest that failures in reaching a functional configuration of the couplings is related to other diseases such as schizophrenia [39], obsessive-compulsive disorder [80], and Parkinson’s. Our model can generalize the dynamical image of these diseases by taking into account learning and chunking dynamics, in order to provide novel insights into treating them.

## Methods

### Transient brain dynamics: Hierarchical chunking

Our overarching hypothesis is that cognitive function in the brain is described by the non-linear interaction of brain “modes”. The number of these modes is assumed much smaller than the number of variables required to describe the state of the brain (*e.g.* membrane potentials, channel states). Backed by recent brain imaging techniques, we follow a top-down approach for identifying the nature of these modes, and how they interact in a transient, robust and scalable fashion to process information [36, 81].

In this context, a mode is defined as a metastable composition of elements from different brain areas that activate coherently to perform a specific cognitive task. Here, we focus on the cognitive task of recalling a sequence, which can be described by the sequential activation of brain modes. In particular, our approach is based on spatiotemporal mental modes that contain metastable states as equilibrium points since it resolves the contradiction by which the system must be robust to noise and, at the same time, sensitive to inputs [52–54].

**Fig 8:**
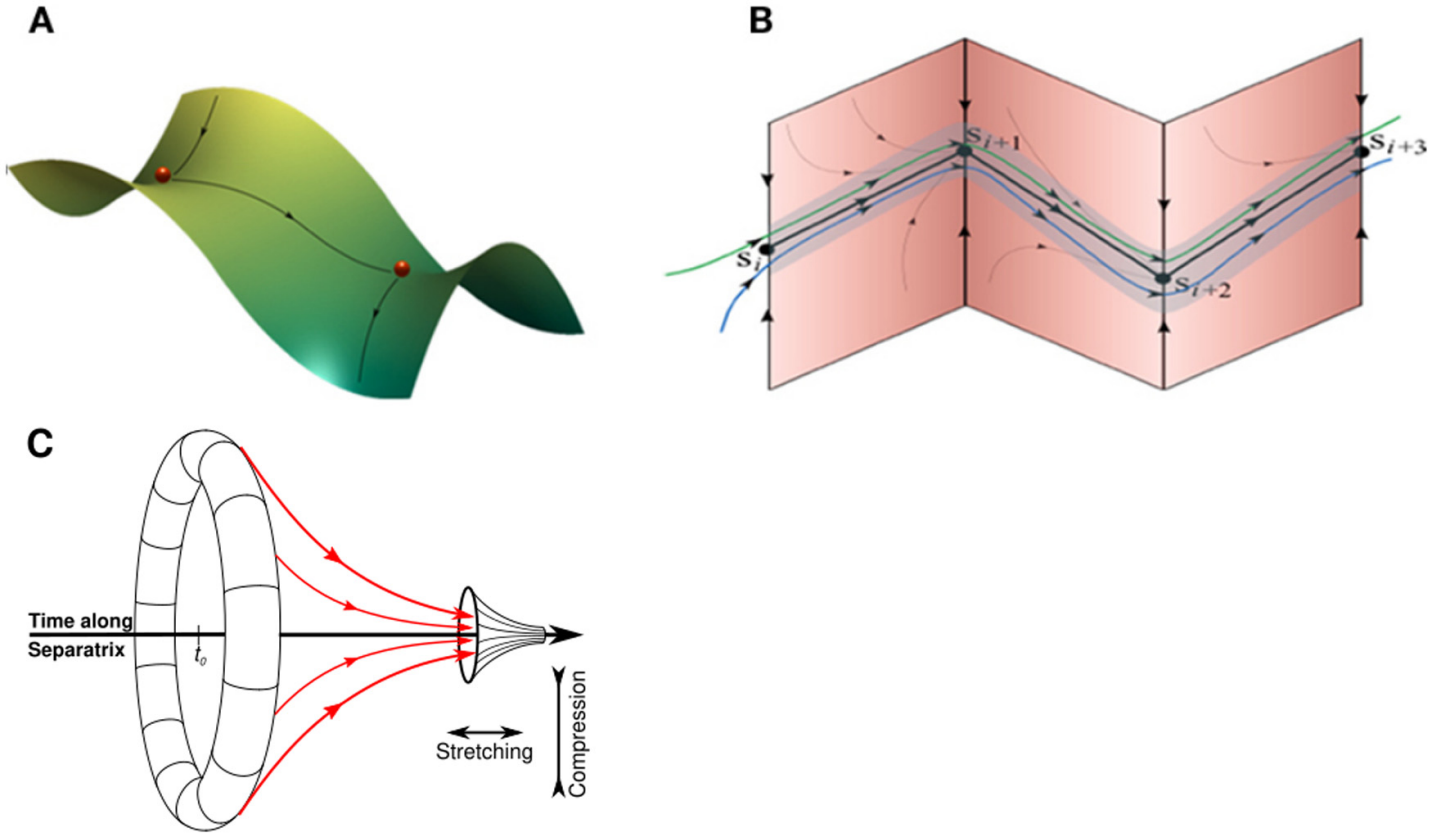
(A) Stable heteroclinic chain with two connected metastable states (B) Stable heteroclinic channel (SHC)—robust sequence of metastable states. Adapted from [82]. (C) Transformation of the phase volume along trajectories in the neighborhood of unstable separatrix in the case when both coupled saddles are characterized by saddle values larger than one.

Metastable states are semi-transient signals that can be represented as saddle nodes. These saddle nodes can be arranged to form a SHC, which consists of a sequence of successive states that are connected through their respective unstable separatrices (Fig 8). Under appropriate parametrizations, namely if the compressing of phase space around the saddle is larger than the stretching and if all saddles in the chain are dissipative, then the trajectories in the neighborhood of the metastable states that form the chain remain in the channel [22].

The GLV dynamics is a canonical model for implementing a SHC [42]:

$$\begin{array}{c} \frac{d}{dt}x_{i}\left(t\right)=x_{i}\left(t\right)(s_{i}\left(t\right)-\sum_{i^{\prime }=1}^{N_{X}}V_{i^{\prime }i}\left(t\right)x_{i}^{\prime }\left(t\right)+\eta_{i}),\;\forall i=1,\cdots ,N \end{array}$$ (5)

The terms *V*_*ii*′_ determine the interaction between the variables *x*_*i*_, and *η*_*i*_ is an additive noise term. This asymmetry in *V*_*ii*′_ installs metastable nodes in the network, which results in successive and temporary winners as in WLC dynamics [23]. The simplicity of this model enables theoretical study of the transient solutions representing sequential competition [42]. The dynamical features of the system Eq (5) extend to a wide class of dynamical systems, known as Kolmogorov models [26]. The biological relevance of these models is confirmed by several previous works [28–30].

The state variables in Eq (5) are modes that represent abstract quantities that do not necessarily map directly or exactly onto individual neuron or populations activities. For instance, [29] show the existence of a SHC in a network of inhibitory Hodgkin Huxley-type (H&H) neurons short-term synaptic depression, despite that the differential equations there differ significantly from Eq (5). Another example is given by [28], which describes the conditions under which the firing rate of leaky Integrate & Fire (I&F) neurons approximately map onto Eq (5).

The hierarchical chunking dynamics is represented by robust transient activity modes at each scale of the hierarchy. The above Eq (5) serves as an elementary building block for each layer of the chunking dynamics. The two-layer chunking dynamics is a GLV system of the form of Eq (1). This model has slight modifications to the one presented in [21], which reflect the necessities for chunk formation during training. Firstly, the polarity of the couplings between the two layers is reversed (in [21] elementary modes inhibit chunking modes). This modification allows the elementary modes to directly drive a CM. Secondly, the synaptic dynamics represented by the dimension *z* are applied to the growth terms of the chunking layer (in contrast to [21], where only inhibitory couplings are subject to synaptic dynamics). The synaptic dynamics helps a single CM to remain active over several items in the stimulus.

### Synaptic plasticity model

The structure of the sequential activity is determined by the connectivity matrix among the respective modes. Within each layer, the amount of asymmetry in the couplings represents an order parameter that controls the dynamical behavior of the network. The inter-layer connections represent the association of the information-carrying items and chunks with the modes. After the presentation of the inputs, the network is run for a consolidation time, and the weights are held fixed to the values reached at the end of this time for recall.

The learning can be understood as the adjustment of this order parameter and the associations in a way that the recall dynamics of the elementary and the chunking modes is consistent with the training sequences.

#### Couplings *P*_*ki*_

The synapses between the PMs and the EMs follow a correlation rule with synaptic scaling [40] Eq (2). The input synapses learn which PMs are associated to a particular pattern. This rule can learn hidden causes of noisy sensory activations in a mixture model [41]. As in [41], we assume that a (unspecified) feedforward inhibition normalizes the intensity of the input patterns such that at steady state, ∑_*k*_ *s*_*k*_ = *C* and ∑_*k*_ *P*_*ki*_ = *C*.

#### Couplings *V*_*ii*′_ and *W*_*jj*′_

The weight update of the coupling between EMs from *i* to *i*′ are dictated by a bistable synaptic plasticity rule with matched potentiation and depression according to Eq (3), where the potentiation and depotentiation terms are:

$$\begin{array}{c} \begin{array}{cc} LTP_{V}\left(x_{i},x_{i^{\prime }}\right)= & \Theta (x_{i}A_{x_{i^{\prime }}}-\theta_{p})\\ LTD_{V}\left(x_{i},x_{i^{\prime }}\right)= & \Theta (x_{i^{\prime }}A_{x_{i}}-\theta_{d})\\ A_{x_{j}}\left(t\right)= & A^{+}\int_{0}^{\infty }K_{A}\left(\Delta \right)x_{j}\left(t-\Delta \right)d\Delta ,\\ A_{x_{i}}\left(t\right)= & A^{-}\int_{0}^{\infty }K_{A}\left(-\Delta \right)x_{j}\left(t-\Delta \right)d\Delta , \end{array} \end{array}$$ (6)

where Θ is the Heaviside (step) function that returns 1 if its argument is positive and 0 otherwise, and *θ*_*p*_, *θ*_*d*_ are constant potentiation and depotentiation thresholds. *A*_*x*_*j*__, *A*_*x*_*i*__ are traces obtained by filtering the activities *x*_*i*_, *x*_*j*_ with the learning window.

*V* ^+^, *V* ^−^, and V* are the fixed points of the bistable learning rule [34].

$$\begin{array}{c} \;V^{+}>V^{*}>V^{-}\geq 0. \end{array}$$

Following this definition, the former are determined to be stable two are stable, while the latter is unstable. Once the weight *V*_*ii*′_ crosses V*, in the absence of stimuli it is attracted towards *V* ^+^ if *V*_*ii*′_ > V* and *V* ^−^ otherwise.

When the activity transitions from one element to another, the synapse along the direction of the transition undergoes depotentiation, while the synapse in the opposite direction undergoes potentiation. At each state transition, this rule depotentiates the inhibitory synapse in the direction of the transition, and potentiates it in the opposite direction.

Initially each unit is associated with a stable fixed point. After a sufficient number of such updates, the stable fixed point becomes a saddle node, where the unstable separatrix leads to the unit associated with the subsequent item in the sequence. The number of updates required for this to occur depends on the magnitude of the synaptic updates, which plays the role of a learning rate. When synaptic potentiation and depression are matched, the weights are modified only when the activities of the modes change (see section 1 of S1 Text, S2 Fig). The same synaptic dynamics apply for the couplings *W*_*jj*′_ among the chunking modes.

#### Couplings *Q*_*ij*_

The chunking layer takes the elementary modes’ activity as its inputs, and associates a group of elementary modes to a CM. The learning rule Eq (4) is a bistable adaptation of Eq (2), where *f*_*Q*_(*Q*) implements the bistable dynamics:

$$\begin{array}{c} f_{Q}\left(Q\right)=\alpha_{Q}\left(Q^{+}-Q_{ij}\left(t\right)\right)\left(Q^{-}-Q_{ij}\left(t\right)\right)\left(Q^{*}-Q_{ij}\left(t\right)\right). \end{array}$$ (7)

The duration of each chunk is strongly dependent on the potentiation and depotentiation scaling factors $\gamma_{p}^{Q}$ and $\gamma_{d}^{Q}$.

A complete analysis of this learning rule is not possible because it involves the non-linear dynamics of both EM and CM. An intuition to the behavior of this rule can be obtained by comparing it to the rule governing *P*. In the case where $\theta_{p}^{Q},\theta_{d}^{Q}=0$, *ϵ*_*H*_ = 0, the rectifying function Θ becomes the identity function since *x*_*i*_ ≤ 0 and *y*_*j*_ ≤ 0. Choosing for clarity *Q* ^−^ = 0, *α*_*Q*_ = 1, $\tau_{Q}=\gamma_{d}^{Q}$, $r=\gamma_{p}^{Q}/\gamma_{d}^{Q}$, the rule becomes:

$$\begin{array}{c} \begin{array}{cc} \frac{d}{dt}Q_{ij}≅ & y_{j}(rx_{i}\left(Q^{+}-Q_{ij}\right)-Q_{ij}), \end{array} \end{array}$$ (8)

which is identical to Eq (2), with the exception of an upper boundary on the weight *Q* ^+^. The conditioning of the stimulus ensures that switches in the chunking layer usually occur only when a new pattern is presented. At each activation of a EM, the active CM can persist or lose competition against another CM. The probability of either event taking place is dictated by the size of the chunk and the initial state of *Q*.

#### Couplings *R*_*ij*_

The chunking modes inhibit the elementary network in a way that the activities of both layers coherently bind to each other. This inhibition is learned with a rule similar to the one above but with swapped boundaries. As a result, when both elementary modes and chunking modes are active, the weight depotentiates (inhibits less), but when only the CM is active, the weight potentiates.

$$\begin{array}{c} \begin{array}{cc} \tau_{R}\frac{d}{dt}R_{ji}=\alpha_{R}f_{R}\left(R_{ji}\right)+ & \gamma_{p}^{R}\left(R^{-}-R_{ji}\right)\Theta \left(x_{i}y_{j}-\theta_{p}^{R}\right)\\ + & \gamma_{d}^{R}\left(R^{+}-R_{ji}\right)\Theta \left(y_{j}-\theta_{d}^{R}\right) \end{array} \end{array}$$ (9)

where,

$$\begin{array}{c} f_{R}\left(R\right)=\alpha_{R}\left(R^{+}-R_{ij}\left(t\right)\right)\left(R^{-}-R_{ij}\left(t\right)\right)\left(R^{*}-R_{ij}\left(t\right)\right). \end{array}$$ (10)

The effect of this rule is to learn a configuration where the EMs associated to the active CM are disinhibited.

### Characterizing sequence recall

At the end of successful training, the network is able to recall the presented sequences. Successful recall is defined when the sequence order is produced with perfect accuracy. However, it occurred that the sequence was reproduced to a reasonable extent (*e.g.* missing elements, sequence reproduced correctly up to certain element). To take into account such events, we used a normalized Levenshtein distance to estimate the quality of the reproduction [83]. This distance computes the number of changes between two sequences (addition, subtraction), normalized by the length of the longest sequence. Note that sequence recall does not characterize chunking since accurate recall can be obtained without learning in the chunking layer.

## Supporting Information

S1 Text Section 1, Details to the learning rule Eq (3). (PDF)

S1 Fig Asymmetric learning windows causes the weight to change when a transition between two units takes place. (TIF)

S2 Fig Network Dynamics Influence Chunking Rate. (TIF)

S3 Fig Chunking rate is modulated by a time-varying bias in the chunking layer. (TIF)

S4 Fig Examples of noisy stimuli. (TIF)
