# Supplementary material for: Learning of Chunking Sequences in Cognition and Behavior
Source: PLoS Comput Biol. 2015 Nov 19;11(11):e1004592. doi: 10.1371/journal.pcbi.1004592 (PMC4652905; doi:10.1371/journal.pcbi.1004592)
Supplement: S1 Text — (PDF) [file pcbi.1004592.s001.pdf]

SI: Learning of chunking sequences in cognition and behavior

Jordi Fonollosa[1,2,◑], Emre Neftci[1,3,◑,*], Mikhail Rabinovich[1]

1 Biocircuits Institute, University of California San Diego, La Jolla, USA
2 Institute for Bioengineering of Catalonia, Barcelona, Spain
3 Department of Cognitive Sciences, University of California Irvine, USA
◑These authors contributed equally to this work.
*** nemre@ucsd.edu**

1 Details to the learning rule Eq. (3)

The update terms are governed by an asymmetric temporal window $K_A(t)$:

$$\begin{aligned} A_{x_j}(t) =& A^+ \int_{-\infty}^{t} K_A(t-s) x_j(s) \mathrm{d}s \\ =& A^+ \int_{0}^{\infty} K_A(\Delta) x_j(t-\Delta) \mathrm{d}\Delta, \\ A_{x_i}(t) =& A^- \int_{-\infty}^{t} K_A(s-t) x_i(s) \mathrm{d}s \\ =& A^- \int_{0}^{\infty} K_A(-\Delta) x_j(t-\Delta) \mathrm{d}\Delta, \end{aligned} \tag{1}$$

where $A^+ > 0$, $A^- > 0$ define the magnitude of the weight update.

For simplicity, we choose an exponential temporal window $K_A(\Delta) = \exp(-|\Delta/\tau_{STDP}|)$ with decay rate $\tau_{STDP} \ll T$. This rule is consistent with the requirement that V_{ij} depotentiates when a transition from x_i to x_j occurs. As long as potentiation and depression are matched, this does not depend critically on this window, as we demonstrate below.

The condition that potentiation and depression are matched can be written:

$$\int_{0}^{\infty} K_A(\Delta) \mathrm{d}\Delta = - \int_{0}^{\infty} K_A(-\Delta) \mathrm{d}\Delta \tag{2}$$

We assume that the sign of $K_A(\Delta)$ is fixed at each side of the $\Delta = 0$ axis:

$$K_A(\Delta < 0) \leq 0, \quad K_A(\Delta > 0) \geq 0 \tag{3}$$

The state typically transitions sharply, such that $x_i(t)$ and $x_j(t)$ are monotonic around the transition times. For a transition, this can be written:

$$\frac{\mathrm{d}}{\mathrm{d}t} x_i(t) \leq 0, \quad \frac{\mathrm{d}}{\mathrm{d}t} x_j(t) \geq 0, \forall t. \tag{4}$$

Under the above assumptions, we show that during a transition from x_i to x_j, V_{ij} depotentiates and V_{ji} potentiates. The weight change is:

$$\begin{aligned} \tau_V \frac{\mathrm{d}}{\mathrm{d}t} V_{ij} =& x_j(t) \int_{0}^{\infty} K_A(s) x_i(t-s) \mathrm{d}s \\ &+ x_j(t) \int_{-\infty}^{0} K_A(s) x_j(t+s) \mathrm{d}s. \end{aligned}$$

Fig. SI 1. A asymmetric learning window (left) causes the weight to change when a transition between two units take place (right).

With a change in sign in the second integral, the above equality can be written:

$$\tau_V \frac{\mathrm{d}}{\mathrm{d}t} V_{ij} = \int_0^\infty x_i(t-\Delta)x_j(t)K_A(\Delta) + x_i(t)x_j(t-\Delta)K_A(-\Delta)\mathrm{d}\Delta.$$

Adding two terms that sum to zero under the integral:

$$\tau_V \frac{\mathrm{d}}{\mathrm{d}t} V_{ij} = \int_0^\infty x_i(t-\Delta)x_j(t)K_A(\Delta) - x_i(t)x_j(t)(K_A(\Delta)+K_A(-\Delta)) + x_i(t)x_j(t-\Delta)K_A(-\Delta)\mathrm{d}\Delta.$$

The matching of the potentiation and depression in Eq. (2) guarantees that the middle terms vanishes.

The terms under the integral can be regrouped as follows:

$$\tau_V \frac{\mathrm{d}}{\mathrm{d}t} V_{ij} = \int_0^\infty x_j(t)(x_i(t-\Delta)-x_i(t))K_A(\Delta) + x_i(t)(x_j(t-\Delta)-x_j(t))K_A(-\Delta)\mathrm{d}\Delta.$$

It is clear that, under the assumptions above (Eq. (2), (Eq. (3)) and (Eq. (4))), the integrand is positive or zero, leading to $\frac{\mathrm{d}}{\mathrm{d}t} V_{ij} \geq 0$. Similarly, $\frac{\mathrm{d}}{\mathrm{d}t} V_{ji} \leq 0$. Fig. 1 illustrates how the asymmetric learning windows causes the weight to change when a transition between two units takes place.

2 Network Dynamics Influence Chunking Rate

The chunking rate is defined as the number of transitions in the chunking layer while a pattern of the sequence is presented in the learning phase. This rate can be modulated, for example by biasing the chunking layer or its auxiliary variables z_k. To illustrate this, we added a global, step-wise varying input to the auxiliary variables z_k, and proceeded with the learning protocol similarly to the experiments in the main text (100 epochs). Results show that a larger number of chunks transition around the steps, and that the input magnitude drastically alters the chunking rate.

3 Learning with Noisy Stimuli

Noisy patterns S'_k were obtained by adding noise to each pattern of the sequence:

$$S'_k[i] = S_k[i] + \max(0, \eta_k[i]), \quad i \in \mathbb{N}, k \in 1, \ldots, M$$

where $\eta_k[i] \sim N(0, \sigma_S)$, and $S_k[i]$ are the original patterns consisting of horizontal bars. The noise term changes from one presentation of the sequence to the other, but it remains constant during the presentation. In the main text, we report the amplitude of the noise as the ratio:

$$\text{Noise amplitude} = \frac{\sum_k^M \langle \eta_k[i] \rangle}{\sum_k^M S_k}$$

where $\langle \cdot \rangle_i$ is the expectation over realizations of $\max(0, \eta_k[i])$. Fig. 3 shows examples of the stimuli with noise amplitudes matching those used in the main text.

Fig. SI 2. Chunking rate is modulated by a time-varying bias in the chunking layer. (Top) We added a global, step-wise varying input b_z to the auxiliary variables z_k. (Middle) Chunking rate computed as the number of transitions in the chunking layer during the presentation of each sequence element, averaged over 60 different runs of the training, and averaged over epochs 50 to 100. The average chunking rate was 0.071 from time 35 to 60, and 1.24 from time 95 to 120. Very few transitions occurred during the phase where b_z was strongly positive, compared to chunking rate .314, when b_z was zero. For strongly negative b_z, chunking is nearly absent, as the chunking layer transitions almost once every presentation of a sequence element (the chunking rate is close to 1). Furthermore, the chunking rate is high at the points where b_z changes, which illustrates how the chunking has a tendency to synchronize with changes in b_z. (Bottom) Illustration of the activity in the chunking layer at trial 50 for all 60 runs. The boundaries of the chunks are clearly located at the time points where b_z changed.

Fig. SI 3. Examples of noisy stimuli, drawn for noise parameters $\sigma_S = 0, .1, .3, .5$, with noise amplitudes estimated at 0%, 38%, 115% and 191%, respectively.

4 Parameters of the learning model

In Tab. 1, we detail all the parameters and values of the learning model so that the dynamics can be reproduced.

Fig. SI 4. An Example of Weight Evolution during Learning, for the run shown in Fig. 6, top right.

Table 1. Parameters of the hierarchical network

N_x	Number of Elementary Mode (EM)	Fig. 2, 3, 4, 5	24
N_y	Number of Chunking Mode (CM)	Fig. 2, 3, 4, 5	3
		All other figures	30
τ_x	Time constant EM	all figures	0.05 au
τ_y	Time constant CM	all figures	$6\tau_x$
τ_z	Synaptic time constant	all figures except Fig. 7	$4\tau_x$
		Fig. 7	.02 – 2
b_x	Growth term EM	all figures, training	0
		all figures, recall	1
b_y	Growth term CM	all figures, training	0
		all figures, recall	.2
$b_z(t)$	Bias term synaptic states	Fig. 2	$-1, 2, 0$
		all other figures	0
C	Total input magnitude of PMs	training, all figures	15
		recall, all figures	0
τ_P	Learning time constant P	all figures	10
τ_V	Learning time constant V	all figures	28.6
τ_W	Learning time constant W	all figures	125
τ_R	Learning time constant R	all figures	333
τ_Q	Learning time constant Q	all figures	125
ϵ_H	Heterosynaptic competition	Fig. 2, 3, 4, 5	0
	Heterosynaptic competition	Fig. 6,7	.001
m_H	Total efferent weight from each EM	Fig. 6,Fig. 7	3
α^V	Scaling of bistable	all figures	.02
	term V	all figures	.02
α^W	Scaling of bistable	all figures	.03
	term W	all figures	.03
θ_d^V,θ_d^W	Depotentation threshold V,W	Fig. 3, 4, 5	0.6
θ_p^V,θ_p^W	Potentation threshold V,W	Fig. 3, 4, 5	0.6
$V(t=0)$,$W(t=0)$	Initial cond. V, W	all figures	off-diagonal 2.1, otherwise 1
V^+,W^+	Positive boundary	Fig. 3, 4, 5	2.55
V^-,W^-	Negative boundary	Fig. 3, 4, 5	0.6
V^*,W^*	Boundary of the basins	Fig. 3, 4, 5	1.77
γ_d^Q	Depotentation factor Q	all figures	.75
γ_p^Q	Potentation factor Q	all figures except Fig. 7	35
		Fig. 7	17.5–105
θ_d^Q	Depotentation threshold Q	all figures	0.2
θ_p^Q	Potentation threshold Q	all figures	0.42
Q^+	Positive boundary	all figures	1
Q^-	Negative boundary	all figures	0
Q^*	Boundary of the basins	all figures	0.4
α^Q	Scaling of bistable	all figures	2.
	term Q	all figures	.02
α^R	Scaling of bistable	all figures	.03
	term R	all figures	1.2
γ_d^R	Depotentation factor R	all figures	0.5
γ_p^R	Potentation factor R	all figures	30
θ_d^R	Depotentation threshold R	all figures	0.2
θ_p^R	Potentation threshold R	all figures	0.25
R^+	Positive boundary	all figures	0.4
R^-	Negative boundary	all figures	0
R^*	Boundary of the basins	all figures	0.1
σ_X	Noise amplitude EM	all figures, training	.02
		all figures, recall	10^{-6}
σ_Y	Noise amplitude CM	all figures, training	.025
		all figures, recall	10^{-6}
