# Supplementary figures and images for: Learning of Chunking Sequences in Cognition and Behavior
Source: PLoS Comput Biol. 2015 Nov 19;11(11):e1004592. doi: 10.1371/journal.pcbi.1004592 (PMC4652905; doi:10.1371/journal.pcbi.1004592)

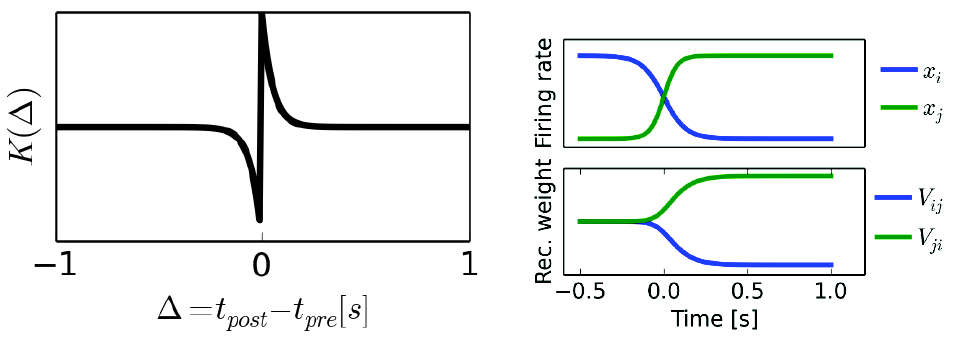

Supplement: S1 Fig — (TIF) [file pcbi.1004592.s002.tif]

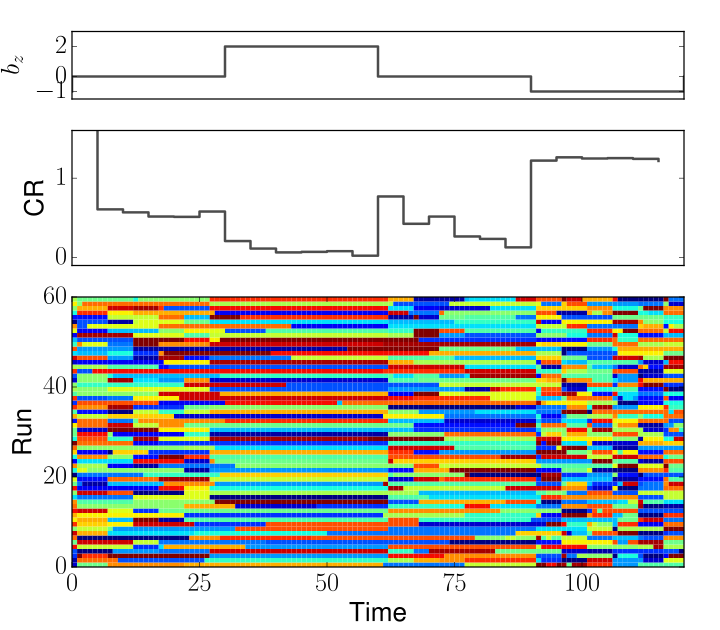

Supplement: S2 Fig — (TIF) [file pcbi.1004592.s003.tif]

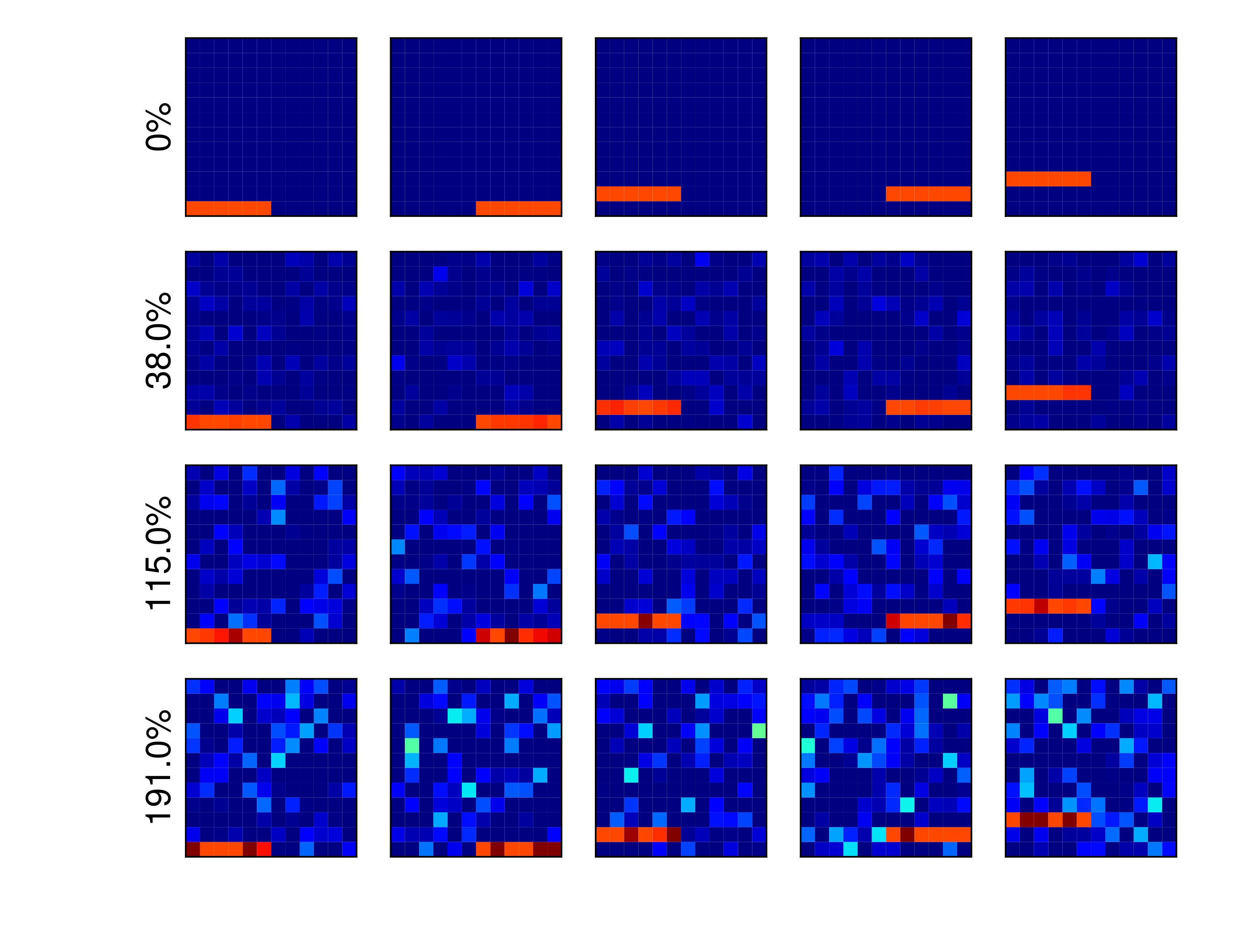

Supplement: S3 Fig — (TIF) [file pcbi.1004592.s004.tif]
